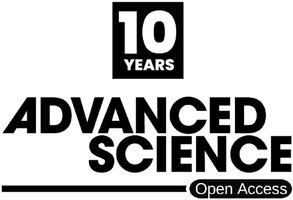# A Decade of Advanced Science

**DOI:** 10.1002/advs.202400981

**Published:** 2024-02-23

We are happy and proud to present you the anniversary issue celebrating 10 years of *Advanced Science*. Time seems to have flown since we came together at the MRS in Boston in 2014 for the launch ceremony. Having started as a tiny journal with a single issue with 5 papers published in 2014, we now look at an established premium gold open access journal that attracted more than 10 000 submissions, and that had a number of full text downloads of 9.7 million in 2023.

Celebrating this important anniversary, we invited our entire editorial board to contribute review articles or perspectives on their favorite topics to this special issue. 26 contributions from 10 different countries, on a wide variety of topics, ranging all the way from chemoproteomics and neuroimmune interaction, to liquid‐crystalline polymer membranes and MXenes for aqueous zinc batteries, make up this exciting issue.

2024 is not only a year of celebration, but also a year of change. As you may have noticed, the editorial team has undergone some change. Our previous deputy editors **Dr. Bo Weng** and **Dr. Xi Wen** have shifted their main focus onto other journals in our portfolio: Bo remains the editor‐in‐chief of *Macromolecular Rapid Communications*, and took on a deputy role in *Advanced Materials*, while Xi recently became the new editor‐in‐chief of *Small Methods*. We continue to work closely with both of them and are grateful for years of hard work and excellent collaboration. **Dr. Ulf Scheffler** moved to an entirely different, but nonetheless important role in Wiley, now leading a team of full‐time experts in publishing ethics. **Dr. Ana Vanessa Jobling** has unfortunately left the company to pursue a new career path in regulatory affairs. All of our former DEs we wish all the best.

Luckily, we have a number of new talented deputy editors, who have joined the team at the same time, giving rise to a stronger team than ever before.

**Dr. Shaoying Cui** joined our team in Nov 2023. She holds a PhD in polymer science and has strong publishing experience in the area of engineering science. **Dr. Xiaoyu Zhang** has already worked on the journal since 2021. With a PhD in Biochemistry and a postdoc in functional genomics, he is a valuable asset for *Advanced Science*, particularly in handling submissions related to biomedical topics. **Dr. Jing Zhu**, who joined the *Advanced Science* team already in 2019, has been serving our materials science journals for more than 12 years and has strong connections to the Chinese research community.

The strong team in China is complemented by our colleagues in Europe. **Dr. Richard Murray**, based in Spain, brings his physics expertise to the team. Richard is also the editor‐in‐chief of the journal *Advanced Intelligent Systems*, with which we will now work together even closer. In the Weinheim office in Germany, we are joined by **Dr. Valentina Lombardo**. She holds a PhD in Materials Science and Nanotechnologies and brings with her five years of experience as a peer review editor for other prestigious journals in our group, like for example *Advanced Materials* and *Small*.

Last, but absolutely not least, I am happy to announce our first deputy editor based in the USA, **Dr. Alanna Gannon**. Alanna's background is on tissue engineering. Before joining *Advanced Science* in 2020, she did a postdoc in regenerative medicine at Harvard. Alanna is based in Boston.

**Dr. Anne Pfisterer** and I will continue to work in our old functions. But also other old friends will stay involved with the journal, like Dr. Duoduo Liang, Dr. Guangchen Xu, Dr. Christine Mayer, Dr. Esther Levy, Dr. Irem Bayindir‐Buchhalter, Dr. Marco Squillaci, and many others. Feel free to approach all of them with feedback or ideas. In addition, multiple new editors have been added to the team in order to deal with the growing number of submissions. Be assured that your papers will be in the best hands.

After the launch ceremony at the MRS in Boston in 2014, we are planning two celebration events for this year. Our deputy editors and other colleagues from our offices in China are planning a symposium on *Biomedical Research* in collaborating with the journal *VIEW*, which will take place in Shanghai in July 2024. The other event will take place in our office in Weinheim, Germany. In collaboration with *Advanced Materials Interfa*ces (celebrating its 10^th^ anniversary as well) and with *Advanced Engineering Materials* (celebrating the 25^th^ anniversary) we will organize a 1 day symposium with some handpicked speakers from our editorial boards and other close friends. More information will come in due course.

For now, I just wanted to thank all board members for contributing to the issue, and my colleague Xi Wen for organizing it. Thanks also to all authors, reviewers, and readers, for 10 years of loyalty to *Advanced Science*.

Enjoy reading our anniversary issue!


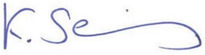


Kirsten Severing

Editor‐in‐Chief